# Some properties for integro-differential operator defined by a fractional formal

**DOI:** 10.1186/s40064-016-2563-0

**Published:** 2016-06-27

**Authors:** Zainab E. Abdulnaby, Rabha W. Ibrahim, Adem Kılıçman

**Affiliations:** Department of Mathematics, Faculty of Science, Universiti Putra Malaysia (UPM), 43400 Serdang, Selangor Malaysia; Faculty of Computer Science and Information Technology, University Malaya, 50603 Kuala Lumpur, Malaysia; Department of Mathematics, Institute for Mathematical Research, Universiti Putra Malaysia (UPM), 43400 Serdang, Selangor Malaysia

**Keywords:** Fractional calculus, Unit disk, Analytic functions, Integral operator, Univalent functions, Fractional differential operator

## Abstract

Recently, the study of the fractional formal (operators, polynomials and classes of special functions) has been increased. This study not only in mathematics but extended to another topics. In this effort, we investigate a generalized integro-differential operator $\mathfrak {J}_{m}(z)$ defined by a fractional formal (fractional differential operator) and study some its geometric properties by employing it in new subclasses of analytic univalent functions.

## Background

The subject of fractional calculus (integral and derivative of any arbitrary real or complex order) has acquired significant popularity and major attention from several authors in various science due mainly to its direct involvement in the problems of differential equations in mathematics, physics, engineering and others for example Baskonus and Bulut (2015), Yin et al. (2015) and Bulut (2016). The fractional calculus has gained an interesting area in mathematical research and generalization of the (derivative and integral) operators and its useful utility to express the mathematical problems which often leads to problems to be solved see Yao et al. (2015), Baskonus (2016) and Kumar et al. (2016). Specifically, it utilized to define new classes and generalized many geometric properties and inequalities in complex domain. In another words, these operators are play an important role in geometric function theory to define new generalized subclasses of analytic univalent and then study their properties. By using the technique of convolution or Hadamard product, Sălăgean (1981) defined the differential operator $\mathcal {D}_{n}$ of the class of analytic functions and it is well known as S$\check{a}$l$\check{a}$gean operator. Followed by Al-Oboudi differential operator see Al-Oboudi (2004). Several authors have used the S$\check{a}$l$\check{a}$gean operator to define and consider the properties of certain known and new classes of analytic univalent functions. We refer here some of them in recent years. Najafzadeh (2010) investigated a new subclass of analytic univalent functions with negative and fixed finitely coefficient based on S$\check{a}$l$\check{a}$gean and Ruscheweyh differential operators. Aouf et al. (2012) gave some results for certain subclasses of analytic functions based on the definition for S$\check{a}$l$\check{a}$gean operator with varying arguments. El-Ashwah (2014) used S$\check{a}$l$\check{a}$gean operator to define a new subclass of analytic functions and derived some subordination results for this subclass in open unit disk. Breaz et al. (2008) investigated a new general integral operator for certain holomorphic functions based on the S$\check{a}$l$\check{a}$gean differential operator and studied some properties for this integral operator on some subclasses of univalent function. Also, Deniz et al. (2012) defined a new general integral operator by considering the Hadamard product and gave new sufficient conditions for this operator to be univalent in $\mathbb {U}$. Breaz et al. (2014) defined two general integral operators $F_{\lambda }(z)$ and $G_{\lambda }(z)$ and investigated some geometric properties for these operators on subclasses of analytic function in open unit disk.

In this paper, we define a generalized mixed integro-differential operator $\mathfrak {J}_{m}(z)$ based on the concept of Breaz integral operator as well as the fractional differential operator and study some their geometric properties on some new subclasses in open unit disk.

## Preliminaries

Let $\mathcal {A}$ denote the class of all functions of the form

1 $$f(z)= z+ \sum _{n=2}^{\infty } a_{n} z^{n}$$

which are analytic function in the open unit disk $\mathbb {U}= \lbrace z: |z|<1\rbrace$ and usually normalized by $f(0)= f^{\prime }(0)-1=0$. Also, let $\mathcal {S}$ be the subclass of $\mathcal {A}$ consisting of functions *f* of form (1) which are univalent in $\mathbb {U}$. We denote by $\mathcal {S}^{*}(\beta )$ and $\mathcal {K}(\beta )$, $0 \le \beta < 1$, the classes of starlike function and convex function in $\mathbb {U}$, respectively. For $f \in \mathcal {A}$, Esa et al. (2016a) introduced the following differential operator $\mathcal {T}^{\alpha ,\delta }: \mathcal {A}\rightarrow \mathcal {A},$

2 $$\begin{aligned} \mathcal {T}^{\alpha ,\delta }f(z) := z + \sum ^{\infty }_{n=2} \frac{\Gamma (\delta +1)}{\Gamma (\alpha +1)} \frac{\Gamma (n+\alpha )}{\Gamma (n+\delta )} a_{n} z^{n} \quad (z\in \mathbb {U}), \end{aligned}$$

for some $(0< \alpha \le 1)$,  $(0 < \delta \le 1)$ and $n \in \mathbb {N}{\setminus }\lbrace 0, 1 \rbrace$. If $\alpha =\delta$ in (2), then we get

$$\mathcal {T}_{z}^{\alpha ,\delta } f(z)= f(z) \quad (z \in \mathbb {U})$$

for more details see Esa et al. (2016b). Now, let define a new fractional differential operator $\mathcal {D}_{\lambda }^{k}:\mathcal {A}\rightarrow \mathcal {A}$ as follows

$$\begin{aligned} \mathcal {D}_{\lambda }^{0}f(z)&= f(z) \\ \mathcal {D}^{1}_{\lambda }f(z)&= (1-\lambda )\mathcal {T}^{\alpha ,\delta }f(z)+\lambda z\left( \mathcal {T}^{\alpha ,\delta }f(z)\right) ^{\prime }, \\ \mathcal {D}_{\lambda }^{k} f(z)&= \mathcal {D}_{\lambda }\left( \mathcal {D}_{\lambda }^{k-1} f(z)\right) \quad (k \in \mathbb {N}, \, z\in \mathbb {U}). \end{aligned}$$

In general, we write

3 $$\begin{aligned} D_{\lambda }^{k}f(z)= z+ \sum _{n=2}^{\infty }\Phi _{n,k}(\alpha ,\delta ,\lambda )a_{n} z^{n}\quad k \in =\lbrace 0,1,2,\ldots \rbrace \end{aligned}$$

where

$$\begin{aligned} \Phi _{n,k}(\alpha ,\delta ,\lambda )=\left[ \frac{\Gamma (\delta +1)}{\Gamma (\alpha +1)}\frac{\Gamma (n+\alpha )}{\Gamma (n+\delta )}(1+(n-1)\lambda )\right] ^{k}. \end{aligned}$$

When $\alpha = \delta$, $\lambda =1$ and $k=1$, we get Sǎlǎgean operator see Sălăgean (1981) and when $\alpha =\delta$, we have Al-Oboudi differential operator see Al-Oboudi (2004). Afterwards, we introduce some new subclasses of $\mathcal {A}$ as follows.

Let $\mathcal {S}^{k}( \lambda ,\phi )$ denote the class of functions $f \in \mathcal {A}$ which satisfies the following condition:

4 $$\begin{aligned} \mathfrak {R}\left\{ \frac{z \mathcal {D}_{\lambda }^{k+1} f(z)}{\mathcal {D}_{\lambda }^{k}f(z)} \right\} > \phi \quad (z \in \mathbb {U}) \end{aligned}$$

for some $0 \le \phi < 1,\, \lambda \ge 0$ and $k \in \lbrace 0,1,\ldots \rbrace$. Let $\mathcal {K}^{k}( \lambda ,\phi )$ denote the class of functions $f \in \mathcal {A}$ which satisfies the following condition

5 $$\begin{aligned} \mathfrak {R}\left\{ 1+ \frac{z \mathcal {D}_{\lambda }^{k+2} f(z)}{\mathcal {D}_{\lambda }^{k+1}f(z)} \right\} > \phi \quad (z \in \mathbb {U}) \end{aligned}$$

for some $0 \le \phi < 1,\, \lambda \ge 0$ and $k \in \lbrace 0,1,\ldots \rbrace$. It is clear that, when $k=0$ in (4) and (5), then we have the well known function classes

$$\begin{aligned} \mathcal {S}^{0}(\lambda ,\phi )=\mathcal {S}^{*}(\phi )\quad \text {and} \quad \mathcal {K}^{0}(\lambda ,\phi )=\mathcal {K}(\phi ). \end{aligned}$$

Further, let $\mathcal {N}^{k}(\lambda ,\psi )$ the subclass of $\mathcal {A}$, consisting of the functions *f*, which satisfies the following

6 $$\begin{aligned} \mathfrak {R}\left\{ 1+ \frac{z \mathcal {D}_{\lambda }^{k+2} f(z)}{\mathcal {D}_{\lambda }^{k+1} f(z)} \right\} < \psi \quad (z \in \mathbb {U}) \end{aligned}$$

and let $\mathcal {M}^{k}(\lambda ,\psi )$ be subclass of $\mathcal {A}$ consisting of the functions *f* which satisfies the following

7 $$\begin{aligned} \mathfrak {R}\left\{ \frac{z \mathcal {D}_{\lambda }^{k+1} f(z)}{\mathcal {D}_{\lambda }^{k}f(z)} \right\} < \psi \quad (z \in \mathbb {U}) \end{aligned}$$

for some $\psi >1$, $\lambda \ge 0$ and $k \in \lbrace 0,1,\ldots \rbrace$. It is obvious that, when $k=0$ in (6) and (7), then we obtain the following classes

$$\begin{aligned} \mathcal {M}^{0}(\lambda ,\psi )= \mathcal {M}(\psi )\quad \text {and} \quad \mathcal {N}^{0}(\lambda ,\psi )=\mathcal {N}(\psi ) \end{aligned}$$

were interested by Owa and Srivastava (2002), Dixit and Chandra (2008) and recently studied by Porwal (2011). Let a function *f* is said to be in the class $\mathcal {K}^{k}\mathcal {L}(\rho ,\varphi )$, if

8 $$\begin{aligned} \mathfrak {R}\left\{ 1+ \frac{z\mathcal {D}_{\lambda }^{k+2}f(z)}{\mathcal {D}_{\lambda }^{k+1}f(z)} \right\} \ge \rho \left| \frac{z\mathcal {D}_{\lambda }^{k+2}f(z)}{\mathcal {D}_{\lambda }^{k+1}f(z)}\right| +\varphi \quad (0 \le \varphi < 1) \end{aligned}$$

for some $\rho ,\, \lambda \ge 0$ and for all $z \in \mathbb {U}$. When $k=0$ in (8), we have the function class studied in Shams and Kulkarni (2004). For $f_{j},\, g_{j} \in \mathcal {A}$ and $\nu _{j},\, \beta _{j}$ be *positive* real numbers, $j=\lbrace 1,2,\ldots ,m \rbrace$, we define the integral operator $\mathfrak {J}_{m}(z):\mathcal {A}^{m} \rightarrow \mathcal {A}$ by

9 $$\begin{aligned} \mathfrak {J}_{m}(z)= \int _{0}^{z}\prod _{j=1}^{m} \left( \frac{\mathcal {D}_{\lambda }^{k}f_{j}(t)}{t}\right) ^{\beta _{j}}\big (\mathcal {D}_{\lambda }^{k+1}g_{j}(t)\big )^{\nu _{j}} dt. \end{aligned}$$

Note that, this integral operator is generalization of the integral operator recently defined by Stanciu and Breaz (2014). Also, the integral operator $\mathfrak {J}_{m}(z)$ is generalizes the following operators defined and investigated by several researchers:

### *Remark 1*

For $k=0$ and $f^{\prime }_{j}= g_{j}^{\prime }, \, j=\lbrace 1,2,\ldots ,m\rbrace$, we have integral operator defined as follows:

$$\begin{aligned} \mathcal {F}_{\nu ,\beta }f(z)= \int _{0}^{z} \prod _{j=1}^{m}\left( \frac{f_{j}(t)}{t}\right) ^{\beta _{j}}\left( {f_{j}^{\prime }(t)}\right) ^{\nu _{j}} dt. \end{aligned}$$

studied and considered by Frasin (2011).

### *Remark 2*

For $k=0$ and $\nu _{j}=0,\, j=\lbrace 1,2,\ldots ,m\rbrace$, we have the following integral operator

$$\begin{aligned} \mathcal {I}_{{m}}(z)= \int _{0}^{z} \prod _{j=1}^{m}\left( \frac{f_{j}(t)}{t}\right) ^{\beta _{j}}dt. \end{aligned}$$

was considered by Breaz and Breaz (2002).

### *Remark 3*

For $k=0$, and $\beta _{j}=0,\, j=\lbrace 1, 2,\ldots , m\rbrace$, we have the integral operator

$$\begin{aligned} \mathcal {I}_{\nu _{m}}(z)= \int _{0}^{z} \prod _{j=1}^{m}\left( {g_{j}^{\prime }(t)}\right) ^{\nu _{j}}dt. \end{aligned}$$

which studied by Breaz et al. (2009). In particular, for $m=1, \nu _{1}=\nu , \beta _{1}=0$ and $g_{1} = g$, we have the integral operator

$$\begin{aligned} \mathcal {I}_{\nu }f(z)= \int _{0}^{z}\left( {g^{\prime }(t)}\right) ^{\nu }dt. \end{aligned}$$

which was considered by Pascu and Pescar (1990).

### *Remark 4*

For $k=0,\, m=1$, $\nu _{1}=0, \beta _{1}=\beta$ and $f_{1}=f$, we have the following operator

10 $$\begin{aligned} \mathcal {I}_{\beta }f(z)= \int _{0}^{z}\left( \frac{f(t)}{t}\right) ^{\beta }dt. \end{aligned}$$

investigated by Miller et al. (1978). In particular, for $\beta =1$, we have the Alexander’s integral operator

11 $$\begin{aligned} \mathcal {I}f(z)= \int _{0}^{z}\frac{f(t)}{t}dt. \end{aligned}$$

which was studied by Alexander (1915).

## Main results

We start our first result.

### **Theorem 1**

*Let*$\nu _{j},\, \beta _{j}$*be positive real numbers, *$j=\lbrace 1,2,\ldots , m\rbrace$. *If*$f_{j} \in \mathcal {M}^{k}(\lambda , \psi _{j} ),\, \psi _{j} > 1$*and*$g_{j} \in \mathcal {N}^{k}(\lambda , \eta _{j}), \,\eta _{j} > 1, j= \lbrace 1,2, \ldots , m \rbrace$, *then the integral operator*$\mathfrak {J}_{m} (z)$*given by* (9) *is in the class*$\mathcal {N}^{k} (\lambda ,\rho )$, *where*

$$\begin{aligned} \rho = 1+\sum _{j=1}^{m}[ \beta _{j}(\psi _{j}-1)+\nu _{j}(\eta _{j}-1)]. \end{aligned}$$

### *Proof*

On successive differentiation of $\mathfrak {J}_{m}(z)$ defined in (9), we obtain

12 $$\begin{aligned} \mathfrak {J}^{\prime }_{m}(z)= \prod _{j=1}^{m} \left( \left( \frac{\mathcal {D}_{\lambda }^{k}f_{j}(z)}{z}\right) ^{\beta _{j}}\left (\mathcal {D}_{\lambda }^{k+1}g_{j}(z)\right )^{\nu _{j}}\right) \end{aligned}$$

and

13 $$\begin{aligned} \mathfrak {J}^{\prime \prime }_{m}(z)&= \sum _{j=1}^{m}\bigg [ \beta _{j}\left( \frac{\mathcal {D}_{\lambda }^{k}f_{j}(z)}{z}\right) ^{\beta _{j}-1} \bigg ( \frac{z\mathcal {D}_{\lambda }^{k+1}f_{j}(z)-\mathcal {D}_{\lambda }^{k}f_{j}(z)}{z^2} \bigg ) \big (\mathcal {D}_{\lambda }^{k+1}g_{j}(z)\big )^{\nu _{j}}\bigg ]\nonumber \\&\quad \times \prod _{\begin{array}{c} \ell =1 \\ \ell \ne j \end{array} }^{m} \left( \left( \frac{\mathcal {D}_{\lambda }^{k} f_{\ell }(z)}{z}\right) ^{\beta _{\ell }} (\mathcal {D}_{\lambda }^{k+1}g_{j}(z))^{\nu _{\ell }} \right) + \sum _{j=1}^{m}\bigg [ \bigg ( \frac{\mathcal {D}_{\lambda }^{k}f_{j}(z)}{z}\bigg )^{\beta _{j}} \nu _j \big (\mathcal {D}_{\lambda }^{k+1}g_{j}(z)\big )^{\nu _{j}-1} \nonumber \\&\quad \times \mathcal {D}_{\lambda }^{k+2}g_{j}(z)\bigg ] \prod _{\begin{array}{c} \ell =1 \\ \ell \ne j \end{array} }^{m} \left( \bigg ( \frac{\mathcal {D}_{\lambda }^{k} f_{\ell }(z)}{z}\bigg )^{\beta _{\ell }} (\mathcal {D}_{\lambda }^{k+1}g_{j}(z))^{\nu _{\ell }} \right) . \end{aligned}$$

By a calculation, we have

14 $$\begin{aligned} \frac{z\mathfrak {J}^{\prime \prime }_{m}(z)}{\mathfrak {J}^{\prime }_{m}(z)}= \sum _{j=1}^{m}\left[ \beta _{j} \left ( \frac{z\mathcal {D}_{\lambda }^{k+1}f_{j}(z)}{\mathcal {D}_{\lambda }^{k}f_{j}(z)}-1 \right ) + \nu _{j} \frac{z\mathcal {D}_{\lambda }^{k+2}g_{j}(z)}{\mathcal {D}_{\lambda }^{k+1}g_{j}(z)} \right] . \end{aligned}$$

The Eq. (14) is equivalent to

15 $$\begin{aligned} \frac{z\mathfrak {J}^{\prime \prime }_{m}(z)}{\mathfrak {J}^{\prime }_{m}(z)} + 1= \sum _{j=1}^{m}\left[ \beta _{j} \left ( \frac{z\mathcal {D}_{\lambda }^{k+1}f_{j}(z)}{\mathcal {D}_{\lambda }^{k}f_{j}(z)}-1 \right ) + \nu _{j} \frac{z\mathcal {D}_{\lambda }^{k+2}g_{j}(z)}{\mathcal {D}_{\lambda }^{k+1}g_{j}(z)} \right] +1. \end{aligned}$$

By calculating the real part of both expressions in (15), we have

$$\begin{aligned} \mathfrak {R}\left\{ \frac{z\mathfrak {J}^{\prime \prime }_{m}(z)}{\mathfrak {J}^{\prime }_{m}(z)} + 1\right\} = \sum _{j=1}^{m}\left[ \beta _{j}\mathfrak {R}\frac{z\mathcal {D}_{\lambda }^{k+1}f_{j}(z)}{\mathcal {D}_{\lambda }^{k}f_{j}(z)}-\beta _{j} + \nu _{j}\mathfrak {R}\frac{z\mathcal {D}_{\lambda }^{k+2}g_{j}(z)}{\mathcal {D}_{\lambda }^{k+1}g_{j}(z)} \right] +1, \\ = \sum _{j=1}^{m}\left [ \beta _{j}\mathfrak {R}\frac{z\mathcal {D}_{\lambda }^{k+1}f_{j}(z)}{\mathcal {D}_{\lambda }^{k}f_{j}(z)}-\beta _{j} + \nu _{j}\mathfrak {R}\left (\frac{z\mathcal {D}_{\lambda }^{k+2}g_{j}(z)}{\mathcal {D}_{\lambda }^{k+1}g_{j}(z)}+1\right )-\nu _{j} \right ]+1. \end{aligned}$$

Since $f_{j} \in \mathcal {M}^{k}(\lambda ,\, \psi _{j}), \psi _{j} > 1$ and $g_{j} \in \mathcal {N}^{k}( \lambda ,\,\eta _{j} ),\,\eta _{j} > 1,\, j=\lbrace 1,2,\ldots ,m\rbrace$, we have

$$\begin{aligned} \mathfrak {R}\left\{ \frac{z\mathfrak {J}^{\prime \prime }_{m}(z)}{\mathfrak {J}^{\prime }_{m}(z)} + 1\right\}&<\sum _{j=1}^{m}[ \beta _{j}\psi _{j}-\beta _{j}+\nu _{j}\eta _{j}-\nu _{j}]+1 \\&< \sum _{j=1}^{m}[ \beta _{j}(\psi _{j}-1)+\nu _{j}(\eta _{j}-1)]+1. \end{aligned}$$

Therefore, $$\mathfrak {J}_{m}(z)\in \mathcal {N}^{k}(\lambda ,\rho )$$, where $$\rho = 1+\sum _{j=1}^{m}[ \beta _{j}(\psi _{j}-1)+\nu _{j}(\eta _{j}-1)]$$.$$\square$$

Let $k=0, m=1$ in Theorem 1, we have

### **Corollary 1**

*Let*$\beta ,\, \nu$*be* positive *real numbers*. *If*$f \in \mathcal {M}(\psi ), \psi > 1$*and*$g \in \mathcal {N}(\eta ), \eta > 1$, *then*

16 $$\begin{aligned} \mathfrak {J}(z)=\int _{0}^{z} \left( \frac{f(t)}{t}\right) ^{\beta } (g^{\prime }(t))^{\nu } dt \end{aligned}$$

*is in the class*$\mathcal {N}(\rho )$, *where*$\rho = 1+ \beta (\psi -1)+\nu (\eta -1)$.

### **Theorem 2**

*Let*$\beta _{j},\,\nu _{j}$*be* positive *real numbers*, $j= \lbrace 1,2,\ldots ,m\rbrace$. *We assume that*$f_{j},\,j=\lbrace 1,2,\ldots ,m\rbrace$*are starlike functions by order*$\frac{1}{\beta _{j}}$*and that is*$f_{j} \in \mathcal {S}^{k}(\lambda ,\,\frac{1}{\beta _{j}})$*and*$g_{j}\in \mathcal {K}^{k}\mathcal {L}(\rho _{j},\, \eta _{j})$, $\rho _{j} \ge 1,\,0 \le \eta _{j} < 1, j=\lbrace 1,2,\ldots ,m \rbrace$. *If*

17 $$\begin{aligned} \sum _{j=1}^{m}[\nu _{j}(1-\eta _{j})+ \beta _{j}]-m <1 \end{aligned}$$

*then*$\mathfrak {J}_{m}(z)$*given by* (9) *is in the class*$\mathcal {K}^{k}(\lambda ,\omega )$*where*

$$\begin{aligned} \omega = 1+ m + \sum _{j=1}^{m}[\nu _{j}(\eta _{j}-1)- \beta _{j}]. \end{aligned}$$

### *Proof*

By following same methods as in Theorem 1, we have

18 $$\begin{aligned} \frac{z\mathfrak {J}^{\prime \prime }_{m}(z)}{\mathfrak {J}^{\prime }_{m}(z)}&= \sum _{j=1}^{m}\left[ \beta _{j} \bigg ( \frac{z\mathcal {D}_{\lambda }^{k+1}f_{j}(z)}{\mathcal {D}_{\lambda }^{k}f_{j}(z)}-1 \bigg ) + \nu _{j} \frac{z\mathcal {D}_{\lambda }^{k+2}g_{j}(z)}{\mathcal {D}_{\lambda }^{k+1}g_{j}(z)} \right] \nonumber \\&= \sum _{j=1}^{m}\left[ \beta _{j} \frac{z\mathcal {D}_{\lambda }^{k+1}f_{j}(z)}{\mathcal {D}_{\lambda }^{k}f_{j}(z)}- \beta _{j} + \nu _{j} \frac{z\mathcal {D}_{\lambda }^{k+2}g_{j}(z)}{\mathcal {D}_{\lambda }^{k+1}g_{j}(z)} \right] , \end{aligned}$$

we can see that, (18) is equivalent to

19 $$\begin{aligned} \frac{z\mathfrak {J}^{\prime \prime }_{m}(z)}{\mathfrak {J}^{\prime }_{m}(z)} +1 = \sum _{j=1}^{m}\left[ \beta _{j} \frac{z\mathcal {D}_{\lambda }^{k+1}f_{j}(z)}{\mathcal {D}_{\lambda }^{k}f_{j}(z)}- \beta _{j} + \nu _{j} \frac{z\mathcal {D}_{\lambda }^{k+2}g_{j}(z)}{\mathcal {D}_{\lambda }^{k+1}g_{j}(z)} \right] +1 \end{aligned}$$

then by taking the real part of (19), we have

20 $$\begin{aligned}&\mathfrak {R}\left\{ \frac{z\mathfrak {J}^{\prime \prime }_{m}(z)}{\mathfrak {J}^{\prime }_{m}(z)} +1\right\} \nonumber \\&\quad =\, \sum _{j=1}^{m}\left[ \beta _{j}\mathfrak {R}\frac{z\mathcal {D}_{\lambda }^{k+1}f_{j}(z)}{\mathcal {D}_{\lambda }^{k}f_{j}(z)}- \beta _{j}+ \nu _{j}\mathfrak {R}\bigg ( \frac{z\mathcal {D}_{\lambda }^{k+2}g_{j}(z)}{\mathcal {D}_{\lambda }^{k+1}g_{j}(z)}+ 1 \bigg ) - \nu _{i} \right] +1, \end{aligned}$$

but $f_{j} \in \mathcal {S}^{k}(\lambda ,\,\frac{1}{\beta _{j}})$, that means $\mathfrak {R}\left\{ \tfrac{\mathcal {D}_{\lambda }^{k+1}f_{j}(z)}{\mathcal {D}_{\lambda }^{k}f_{j}(z)}\right\} > \frac{1}{\beta _{j}}$ and $g_{j} \in \mathcal {K}^{k}\mathcal {L}(\rho _{j},\eta _{j})$, $\rho _{j} \ge 0$ and $0 \le \eta _{j}< 1$,  $j=\lbrace 1,2,\ldots ,m\rbrace$, from (20), we have

$$\begin{aligned} \mathfrak {R}\left\{ \frac{z\mathfrak {J}^{\prime \prime }_{m}(z)}{\mathfrak {J}^{\prime }_{m}(z)} +1\right\}&>\sum _{j=1}^{m}\left[ 1 - \beta _{j}+ \nu _{j}\left (\rho _{i}\left | \frac{z\mathcal {D}_{\lambda }^{k+2}g_{j}(z)}{\mathcal {D}_{\lambda }^{k+1}g_{j}(z)}\right | + \eta _{j} \right )-\nu _{j} \right] +1 \\&> 1+ m -\sum _{j=1}^{m}\beta _{j} +\sum _{j=1}^{m} \nu _{j}\rho _{i}\left | \frac{z\mathcal {D}_{\lambda }^{k+2}g_{j}(z)}{\mathcal {D}_{\lambda }^{k+1}g_{j}(z)}\right | + \sum _{j=1}^{m}\nu _{j}(\eta _{j}-1). \end{aligned}$$

Since,   $\nu _{j}\,\rho _{j}\,\big | \tfrac{z\mathcal {D}_{\lambda }^{k+2}g_{j}(z)}{\mathcal {D}_{\lambda }^{k+1}g_{j}(z)}\big |>0$, we have

$$\begin{aligned} \mathfrak {R}\left\{ \frac{z\mathfrak {J}^{\prime \prime }_{m}(z)}{\mathfrak {J}^{\prime }_{m}(z)} +1\right\}&>1+ m - \sum _{j=1}^{m}\beta _{j} + \sum _{j=1}^{m}\nu _{j}(\eta _{j}-1),\\&> 1+ m + \sum _{j=1}^{m}[\nu _{j}(\eta _{j}-1)- \beta _{j}]. \end{aligned}$$

By using the condition in (17), we have that $\mathfrak {J}_{m}(z) \in \mathcal {K}^{k}(\lambda ,\omega )$, where

$$\begin{aligned} \omega = 1+ m + \sum _{j=1}^{m}[\nu _{j}(\eta _{j}-1)- \beta _{j}]. \end{aligned}$$

$$\square$$

By setting $k=0$ and $m=1$ in Theorem 2, we have the following result.

### **Corollary 2**

*Let*$\beta , \nu$*bepositive real numbers, We assume that*$f \in \mathcal {S}^{*}(\frac{1}{\beta })$, $g \in \mathcal {K}\mathcal {L}(\rho ,\, \eta ), \rho \ge 0$*and*$0 \le \eta < 1$. *If*

$$\begin{aligned} \beta + \nu (1-\eta ) < 2 \end{aligned}$$

then the integral operator

$$\begin{aligned} \mathfrak {J}(z)=\int _{0}^{z}\left( \frac{f(t)}{t}\right) ^{\beta }( g^{\prime }(z))^{\nu } dt \end{aligned}$$

is in the class $\mathcal {K}(\omega )$ where  $\omega =2+\nu (\eta -1)-\beta .$

## Conclusion

In geometric function theory, we defined and studied a new integro -differential operator $\mathfrak {J}_{m}(z)$, with a new classes of analytic and univalent functions. This operator is generalized and modified recent various fractional differential operators.
